# A Community Resource Benchmarking Predictions of Peptide Binding to MHC-I Molecules

**DOI:** 10.1371/journal.pcbi.0020065

**Published:** 2006-06-09

**Authors:** Bjoern Peters, Huynh-Hoa Bui, Sune Frankild, Morten Nielsen, Claus Lundegaard, Emrah Kostem, Derek Basch, Kasper Lamberth, Mikkel Harndahl, Ward Fleri, Stephen S Wilson, John Sidney, Ole Lund, Soren Buus, Alessandro Sette

**Affiliations:** 1 La Jolla Institute for Allergy and Immunology, San Diego, California, United States of America; 2 Center for Biological Sequence Analysis, BioCentrum-DTU, Technical University of Denmark, Lyngby, Denmark; 3 Department of Experimental Immunology, Institute of Medical Microbiology and Immunology, University of Copenhagen, Copenhagen, Denmark; Columbia University, United States of America

## Abstract

Recognition of peptides bound to major histocompatibility complex (MHC) class I molecules by T lymphocytes is an essential part of immune surveillance. Each MHC allele has a characteristic peptide binding preference, which can be captured in prediction algorithms, allowing for the rapid scan of entire pathogen proteomes for peptide likely to bind MHC. Here we make public a large set of 48,828 quantitative peptide-binding affinity measurements relating to 48 different mouse, human, macaque, and chimpanzee MHC class I alleles. We use this data to establish a set of benchmark predictions with one neural network method and two matrix-based prediction methods extensively utilized in our groups. In general, the neural network outperforms the matrix-based predictions mainly due to its ability to generalize even on a small amount of data. We also retrieved predictions from tools publicly available on the internet. While differences in the data used to generate these predictions hamper direct comparisons, we do conclude that tools based on combinatorial peptide libraries perform remarkably well. The transparent prediction evaluation on this dataset provides tool developers with a benchmark for comparison of newly developed prediction methods. In addition, to generate and evaluate our own prediction methods, we have established an easily extensible web-based prediction framework that allows automated side-by-side comparisons of prediction methods implemented by experts. This is an advance over the current practice of tool developers having to generate reference predictions themselves, which can lead to underestimating the performance of prediction methods they are not as familiar with as their own. The overall goal of this effort is to provide a transparent prediction evaluation allowing bioinformaticians to identify promising features of prediction methods and providing guidance to immunologists regarding the reliability of prediction tools.

## Synopsis

In higher organisms, major histocompatibility complex (MHC) class I molecules are present on nearly all cell surfaces, where they present peptides to T lymphocytes of the immune system. The peptides are derived from proteins expressed inside the cell, and thereby allow the immune system to “peek inside” cells to detect infections or cancerous cells. Different MHC molecules exist, each with a distinct peptide binding specificity. Many algorithms have been developed that can predict which peptides bind to a given MHC molecule. These algorithms are used by immunologists to, for example, scan the proteome of a given virus for peptides likely to be presented on infected cells. In this paper, the authors provide a large-scale experimental dataset of quantitative MHC–peptide binding data. Using this dataset, they compare how well different approaches are able to identify binding peptides. This comparison identifies an artificial neural network as the most successful approach to peptide binding prediction currently available. This comparison serves as a benchmark for future tool development, allowing bioinformaticians to document advances in tool development as well as guiding immunologists to choose good prediction algorithm.

## Introduction

Cytotoxic T lymphocytes of the vertebrate immune system monitor cells for infection by viruses or intracellular bacteria by scanning their surface for peptides bound to major histocompatibility complex (MHC) class I molecules (reviewed in [1]). The presented peptides are generated within the cells during the degradation of intracellular proteins. Cells presenting peptides derived from nonself proteins, such as viruses or bacteria, can trigger a T-cell immune response leading to the destruction of the cell. Likewise, this peptide presentation mechanism is utilized to detect cancerous cells [2] and—when malfunctioning—is implicated in several autoimmune diseases [3].

Peptides bound to MHC molecules that trigger an immune response are referred to as T-cell epitopes. Identifying such epitopes is of high importance to immunologists, because it allows the development of diagnostics, evaluation of the efficacy of subunit vaccines, and even the development of peptide-based vaccines. Many computational algorithms have been created to predict which peptides contained in a pathogen are likely T-cell epitopes [4–25]. Such tools allow for the rapid scan of the proteome of a pathogen, and are being widely used in the immunological community. Many of them are freely available on the internet.

Multiple factors influence whether a peptide contained in the proteome of a pathogen is an epitope (i.e., whether it can trigger an immune response). For T-cell epitopes, the most selective requirement is the ability to bind to an MHC molecule with high affinity. Binding is also the most straightforward factor to characterize experimentally as well as model computationally, since the ability of a peptide to bind an MHC molecule is encoded in its primary amino acid sequence. Predictions for peptide cleavage by the proteasomal and peptide transport by the transporter associated with antigen presentation (TAP) have been developed as well [8,15,26–31], but the influence of these processes on peptide recognition is more difficult to model, as alternative pathways exist [32–35], and the generation of precursor peptides has to be taken into account.

An essential step in developing prediction tools is to gather a set of experimental training data. This is typically either derived from in-house experiments, published literature, or querying one or more of the specialized databases containing epitope-related information such as Syfpeithi [13], MHCBN [36], AntiJen [37], HLA Ligand [16], FIMM [38], and our own project, the Immune Epitope Database (IEDB) [39,40]. However, these databases are not primarily designed with tool developers in mind, and extracting a consistent set of training data can be a nontrivial exercise. Furthermore, algorithm developers are not always aware of the implications of mixing data from different experimental approaches, such as T-cell response, MHC ligand elution, and MHC binding data.

Even within a single assay category, such as MHC binding experiments, mixing data from different sources without further standardization can be problematic. When we gathered data from the literature to establish the IEDB, we found 200 peptides with MHC binding reported in three or more sources. Out of these, 37 had conflicting classifications into both binding and nonbinding peptides. This is most often due to the fact that with new studies and assay systems, new criteria are set for what is deemed positive. To merge different datasets, it would therefore be highly beneficial to know how measurements from different assays compare quantitatively.

Having assembled a set of training data, the next step is to choose a prediction method, such as a certain type of artificial neural network (ANN), hidden Markov model, or regression function, which can generate a prediction tool from a set of training data. (Throughout this manuscript, we distinguish between the prediction *tool,* such as a trained neural network that can be used to make predictions, and the *method* used to generate it.) With a newly generated prediction tool, the next essential step is to compare the performance with previously published work. However, there are no accepted standards for testing and evaluating newly developed tools that would allow researchers to unequivocally communicate the advances made with a new tool to the bioinformatics and immunological community. This has lead the majority of experimental immunologists to rely on established predictions such as those provided by bimas [10] and syfpeithi [13], or to stick with methods established in their laboratories.

The goal of this work is to provide a community platform that aids in the generation and evaluation of epitope prediction tools. We focus on MHC class I binding predictions, for which the most experimental data are available, and good prediction methods are best defined. The platform consists of two main components. One is the assembly of a large and consistent dataset of MHC–peptide binding measurements that is to be made publicly available for training and testing purposes. Benchmark predictions of publicly available tools for this set are provided. The second component is an expandable automated framework for the generation and evaluation of prediction methods. This allows scientists to add their prediction methods for a fully transparent side-by-side comparison with other prediction methods in which both training and testing data are controlled. We employed this framework to compare three prediction methods utilized by us in-house, an ANN [24,41], and two matrix-based prediction methods: average relative binding (ARB [5]) and the stabilized matrix method (SMM [42]).

## Results

### Assembling the Dataset

We have collected measured peptide affinities to MHC class I molecules from two sources: the group of Alessandro Sette at the La Jolla Institute for Allergy and Immunology [43], and the group of Søren Buus at the University of Copenhagen [44]. The assays used by the two groups are different in several aspects, such as the indicator used to detect binding (bound radioactive ligand vs. quantitative enzyme-linked immunosorbent assay), what is detected (competitive binding vs. refolding), the way the MHC molecules are prepared (isolated from homozygous cell lines vs. recombinant MHC), and the purity of peptides used (crude synthesis vs. purified peptide). The type of data generated, however, is the same: each peptide gets assigned an affinity to a given MHC allele in terms of IC_50_/EC_50_ nM (for brevity, we will refer to EC_50_ as IC_50_ in the following). Peptides with an affinity worse than the experimental sensitivity threshold are assigned an upper limit of detectable IC_50_ (Sette: >50,000 nM or higher; Buus: >20,000 nM). If affinities for the same peptide to the same MHC molecule were recorded in multiple assays, the geometric mean of the IC_50_ values was taken as the consensus value in the final dataset.

The final dataset is heterogeneous with regard to the peptide sequence tested for binding to each allele. On average, 84% of the peptides in each dataset differed in at least two residues with every other peptide in the set. No additional homology reduction was performed on the peptide sequences, because this should be done by the tool developers, who may prefer to use different homology-reduction approaches that are best optimized for their specific methods. Our purpose is to provide a complete training dataset to the public.

Table 1 gives an overview of the data, comprising 48,828 recorded affinities of peptides for a total of 48 different mouse, human, macaque, and chimpanzee MHC class I alleles. The amount of data available per allele varies greatly from 51 recorded affinities for 11-mer peptides binding to the mouse MHC allele H-2 K^k^ to 3,089 affinities for 9-mer peptides binding to the well-studied human allele HLA-A*0201. The entire dataset is available for download at http://mhcbindingpredictions.immuneepitope.org.

**Table 1:**
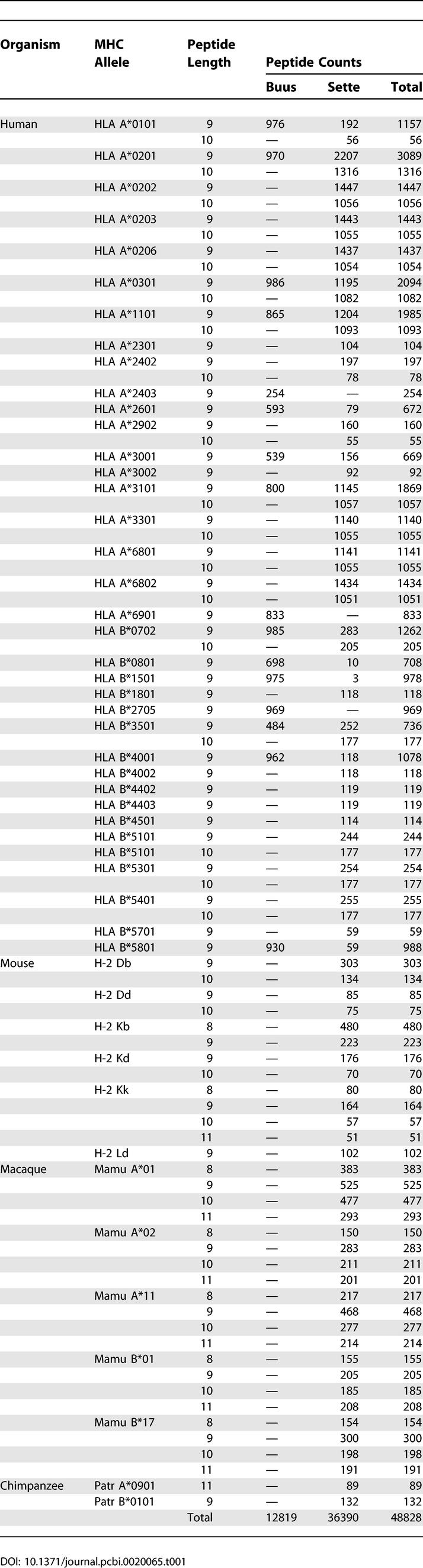
Dataset Overview

Compared to other public databases, this is a much more homogenous set of data, as all of it was generated in one of only two assay systems. At the same time, the amount of data in our set is much greater than what was previously available. By comparison, the largest set of quantitative peptide affinities to MHC class I molecules currently available is found in the AntiJen database, which contains 12,190 datapoints that are compiled from the literature and were derived with a large variety of different assays.

**Figure 1:**
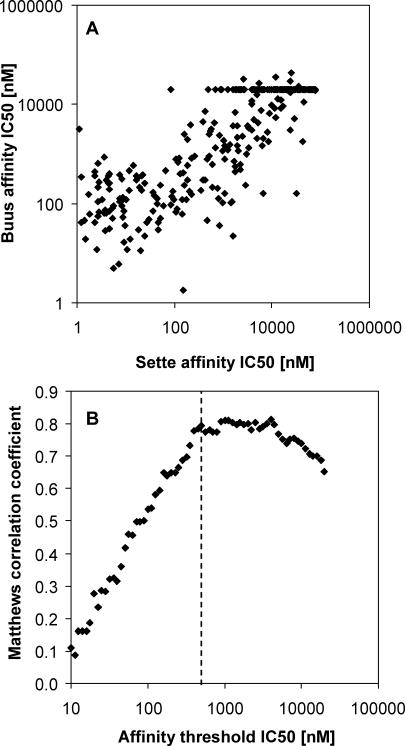
Comparability of the Binding Affinities between Assays (A) Scatter plot comparing measured affinities for peptides to MHC recorded in the Buus (*y*-axis) and Sette (*x*-axis) assay systems. (B) The agreement between experimental classifications of peptides as binders/nonbinders at different affinity thresholds (*x*-axis) is measured by the Matthews correlation coefficient (*y*-axis). The dashed lines indicates the IC_50_ = 500 nM cutoff commonly used for classifying peptides into binders and nonbinders, which is used in the ROC analysis.

To evaluate how comparable the IC_50_ values between the two assays are, we have exchanged sets of peptides and experimentally measured their affinity to MHC alleles available in both assay systems. The scatterplot in Figure 1A shows that there is good agreement between the two assays for intermediate- and low-affinity peptides, less so for high-affinity peptides. To quantify the level of agreement between the two assays, we utilized Matthew's correlation coefficients as a measure of classification agreement, which yield values of 1.0 for perfect agreement and 0.0 for uncorrelated classifications (Figure 1N). For IC_50_ higher than 150 nM, the correlation coefficient is consistently above 0.65, indicating good agreement between the two assays. Conveniently, at the IC_50_ = 500 nM cutoff, which is commonly used to classify peptides into binders (IC_50_ < 500 nM) or nonbinders (IC_50_ ≥ 500 nM) [45], the two assays show very good agreement with a Matthew's correlation coefficient of 0.80.

For peptides with high affinities of IC_50_ = 50 nM or better, the two assays show much less agreement, with correlation coefficients below 0.37. One explanation consistent with the observed differences is that for very-high–affinity peptides, determining K_D_ based on IC_50_ values may no longer be reliable as the concentration of MHC molecules is no longer negligible compared to the peptide concentration used for saturation (also known as “ligand depletion”) [46].

The assay comparisons presented herein provide an example of how pooling experimental data from different sources without additional validation can be problematic; the differences encountered between the measurements of the two closely related assays here are small compared to the differences found when curating from the literature, which is derived by a multitude of different experimental approaches.

### Evaluating Prediction Methods

We used this dataset to compare the performance of three prediction methods currently used in-house in our labs: the ARB [5] and SMM [42] methods generate scoring matrices, while the ANN [41] method generates an artificial neural network. All three methods predict the quantitative affinity of a peptide for an MHC molecule. At this time, the ANN method has only been applied to the prediction of peptides of length nine. We are currently working on expanding this algorithm to make prediction for different lengths possible, but in the comparison presented here, we intentionally did not modify any of the three prediction methods from their previously published implementations.

With the dataset described above, we used five-fold cross-validation to generate and evaluate predictions for each of the three methods. For each allele and peptide length combination, the available data were split into five equally sized sets, of which four were used to generate a prediction tool (i.e., a matrix or a neural network). The tool generated was then used to predict the affinities of the peptides in the left-out set. Repeating this five times, each peptide in the original dataset was assigned a predicted score.

**Figure 2:**
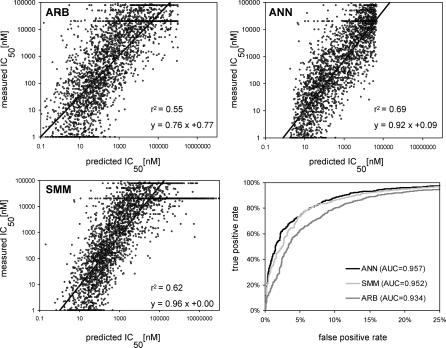
ARB, SMM, and ANN Predictions for HLA-A*0201 The first three panels depict scatter plots of the predicted binding scores (*x*-axis) against the measured (*y*-axis) binding affinities of 3,089 9-mer peptides to HLA-A*0201. The predictions were obtained in five-fold cross-validation using the ARB/SMM/ANN prediction methods, respectively. In each plot, a linear regression on a logarithmic scale was performed, and the corresponding regression equation and *r^2^* values are given. The bottom right panel contains an ROC analysis of the same data, evaluating how well the three methods can classify peptides into binders (IC_50_ < 500 nM) and nonbinders. The AUC, which evaluates prediction quality, is given for each method.

Figure 2 depicts scatter plots comparing the measured affinities of 3,089 nonamer peptides to HLA-A*0201 with their predicted scores for the three methods. The expected positive correlation between predicted and measured affinities was observed for each method. Note that a large fraction of measured affinities have their value set to the upper detection limit (>20,000 nM), and therefore appear as horizontal lines of dots in the scatter plots. Also note that the three methods handle very high and low predicted values differently. The ANN predictions are limited to values between 1 and 50,000 nM and the ARB predictions are similarly capped at 10^6^ nM, while the SMM predictions are not capped at all, which can lead to predictions outside of the experimentally observable range.

To quantitatively compare prediction quality, we calculated linear correlation coefficients between predicted and measured affinities on a logarithmic scale. For this calculation, all peptides with measured affinities at the upper detection limit were ignored. The resulting correlation coefficients are ARB = 0.55, SMM = 0.62, and ANN = 0.69, making the ANN predictions the best in a statistically significant manner (*p* < 0.05 using a *t* test for correlation coefficients drawn from the same sample [47]). The corresponding linear regression curves are included in Figure 2.

An alternative measure of prediction quality is a receiver operating characteristic (ROC) analysis. This evaluates how well the predicted scores classify peptides into binders (experimental IC_50_ < 500 nM) and nonbinders (experimental IC_50_ ≥ 500 nM) by plotting the rate of true positive classifications as a function of the false-positive classifications for all possible cutoffs in the prediction output. The overall quality of the prediction is measured by the area under the ROC curve (AUC), which is 1.0 if the prediction is perfect and 0.5 if it is random. This metric has the advantage that (1) it is invariant to different scales of the prediction output and only slightly affected by prediction caps; (2) it is more robust against outliers than a regression analysis; and (3) all measurements including peptides without quantitative affinities (e.g., >20,000 nM) can be utilized. Also, our two experimental sources show very good agreement at the IC_50_ = 500 nM cutoff (Figure 1). This means that an ROC analysis at this cutoff is less prone to artifacts introduced by pooling the two sets of data than the regression analysis.

Figure 2 presents ROC curves for the three methods. Comparing classifications with the same rate of false positives, the ANN predictions always have an equal or higher rate of true positives than the SMM predictions, which in turn outperform the ARB predictions. This is reflected in the AUC values of ARB = 0.934, SMM = 0.952, and ANN = 0.957, which again shows the ANN predictions to be significantly better than the others (*p* < 0.05 using a paired *t* test on AUC values generated by bootstrap as described in Materials and Methods).

We repeated the same analysis for all MHC alleles and peptide lengths for which we have binding data available. Table 2 shows the AUC values for each method. Comparing only the predictions for 9-mer peptides, where all three methods were available, shows that the ANN predictions are the best in 30 cases, the SMM predictions in 16, and the ARB predictions in zero cases. The differences between the predictions of the three methods is statistically significant (ARB < SMM < ANN) as evaluated by a paired *t* test and a Wilcoxon signed-rank test (both with *p* < 0.05). Comparing the prediction performance utilizing correlation coefficients instead of AUC values gives very similar results, as does repeating the ROC analysis with classification cutoffs of IC_50_ = 50 and 5,000 nM instead of 500 nM (unpublished data).

**Table 2:**
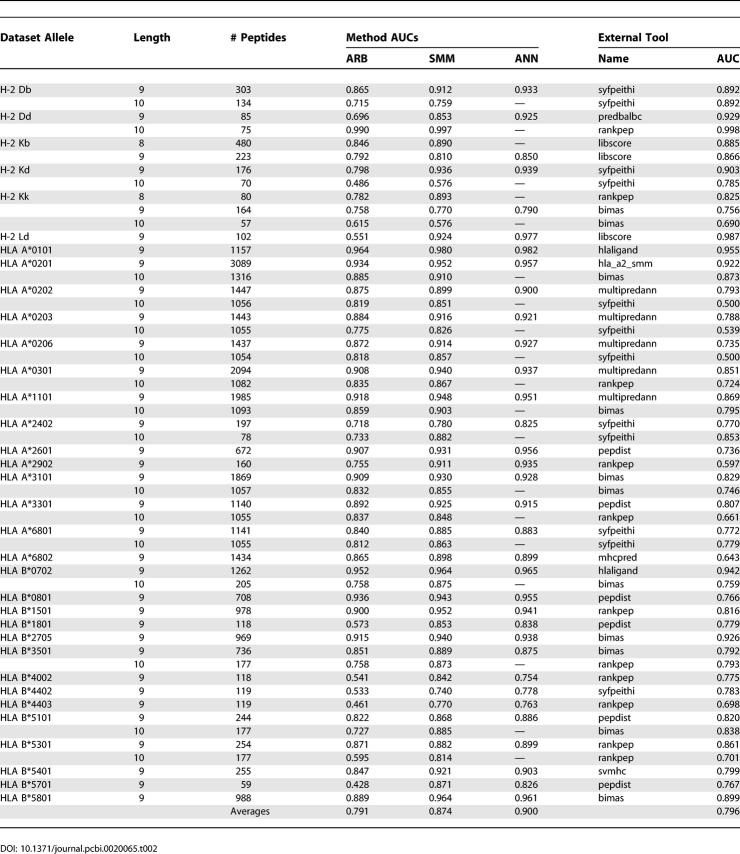
Overview of Prediction Performance as Measured by AUC Values

**Figure 3:**
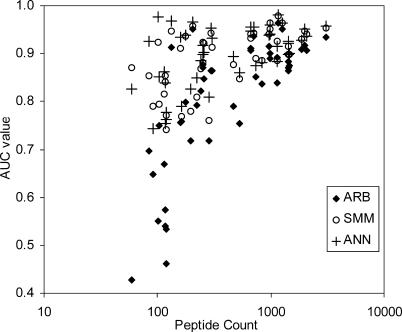
Prediction Performance as a Function of Training Set Size For all datasets for which predictions with all three methods could be made, the AUC values obtained with the three prediction methods are included in the graph (*y*-axis). The *x*-axis gives the number of peptide affinities in each training set.

It is commonly assumed that scoring matrices can be useful for smaller datasets, while neural networks should outperform them if large training datasets are available [38,48], as they can model higher-order correlations that require a large dataset to be estimated precisely. To analyze how well this is reflected in our results, we plotted the AUC values of each method as a function of peptides in the training set (Figure 3). For datasets with less than 300 peptides available, the ANN method performs best, outperforming the SMM method in 16 of 23 cases. Interestingly, this ratio does not increase for datasets containing more than 300 peptides, for which it outperforms the SMM method in 14 of 23 cases. This indicates that the primary limiting factor for the performance of the SMM method is not its inability to model higher-order correlations, which would have resulted in an increasing performance gap for larger datasets. The same is true for the ARB method, as it gains the most from increasing amounts of data, which again indicates that the matrix representation per se is not the primary reason for its underperformance; rather, it is the accuracy of the determined matrix values that improves as the amount of data increase.

### Comparison with Publicly Available Prediction Tools

As far as possible, we also wanted to compare our results with other existing predictions. In October and November 2005, we retrieved predictions from all tools known to us to be freely accessible on the internet for all the peptides in our dataset. Only servers that (1) provided predictions for the alleles in our dataset; (2) were available during that time; and (3) did not specifically disallow the use of automated prediction retrieval were taken into account. This included the following 16 tools: arbmatrix [5], bimas [10], hlaligand [16], hla_a2_smm [12], libscore [17], mappp [8], mhcpathway [18], mhcpred [7], multipred [19], netmhc [20,24,41], pepdist [22], predbalbc [23], predep [21], rankpep [14], svmhc [6], and syfpeithi [13]. We always used the default parameter settings for each tool, and we used the immediate tool output as seen by a user. No attempts were made to optimize the results for any tool once meaningful predictions could be retrieved. We are aware that this may lead us to underestimate the performance of some tools (e.g., svmhc provides an alternative output format with quantitative values for nonbinding predictions) that we have initially overlooked.

**Figure 4:**
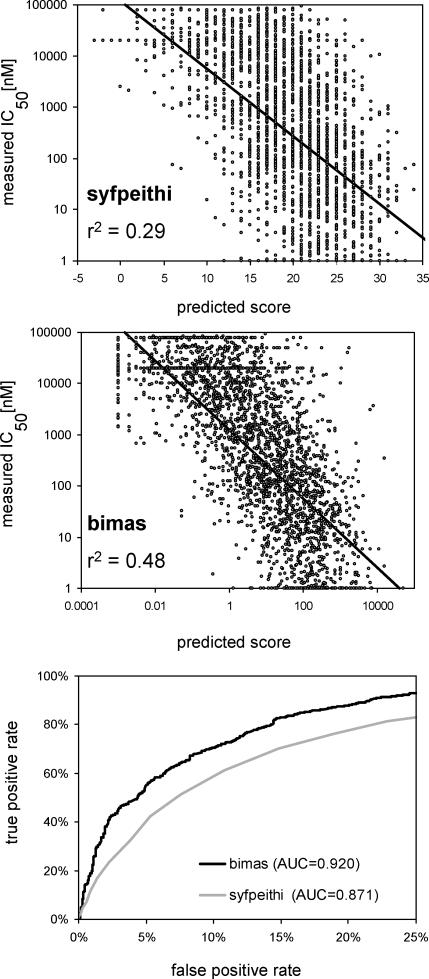
Syfpeithi and Bimas Predictions for HLA-A*0201 The top two panels contain scatter plots of the predicted binding scores (*x*-axis) against the measured binding affinities (*y*-axis) for all 3,089 9-mer peptides binding to HLA-A*0201 in our database. Both bimas and syfpeithi do not predict IC_50_ values, but have output scales in which high scores indicate good binding candidates. Therefore, the regression curves are inverted. The bottom panel contains an ROC analysis of the same data with the classification cutoff of 500 nM.

Figure 4 shows predictions retrieved for peptides binding to HLA-A*0201 from two of the most established prediction methods, bimas and syfpeithi. The prediction output of these two methods are not IC_50_ values, but estimates of half-life of dissociation in the case of bimas, and an integer score for the syfpeithi predictions. In both cases, higher predictions correspond to better binders, which here means lower IC_50_ values. An ROC analysis of these predictions is also shown in Figure 4, which gives AUC values of 0.920 for bimas and 0.871 for syfpeithi. A sample collection of AUC values for more alleles and other external prediction tools is shown in Table 3. For each allele and peptide length, the tool with the highest AUC value among the set of external tools is listed in the last two columns of Table 2. The complete listing for all external tools is contained in Table S1, which also contains the URLs from which predictions were obtained.

**Table 3:**
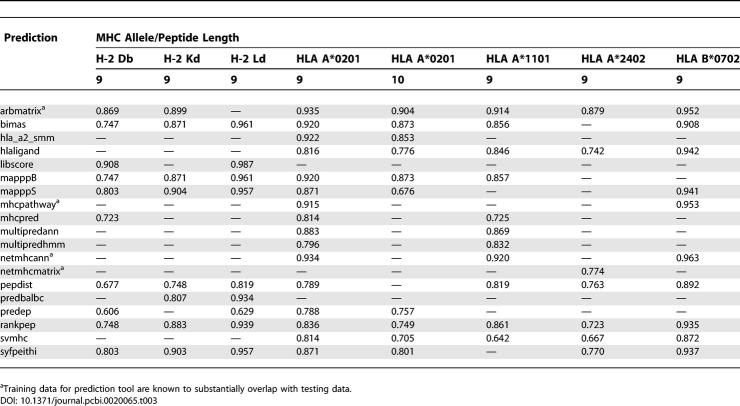
Prediction Quality of Tools Available Online

It has to be stressed that this analysis does not fairly judge the performance of external predictions in all cases. For example, some methods such as syfpeithi do not aim to specifically predict peptide binding to MHC molecules, but rather naturally processed peptide ligands. Also, the amount and quality of training data available to each method are divergent, which disadvantages methods with little access to training data. In contrast, some tools were generated with an appreciable fraction of data that is used here for testing. Such nonblind tool generation leads to an overestimation of performance. These tools are marked with an asterisk (*) in Table 3, and are excluded from Table 2.

In light of the above caveats, we focus on successful external predictions. In total there were 54 allele/peptide length combinations for which we had at least one external prediction tool available (Table 2). For eight of these 54 combinations, one of the external tools performed better than all three of our methods. These eight tools were derived from bimas (one instance), libscore (two instances), predbalbc (one instance), rankpep (one instance), and syfpeithi (three instances). For seven out of eight of these tools, the corresponding datapoints available for training in our affinity set are comparably few (<140), which may explain the low performance of our three methods for these sets. For 9-mers binding to H-2 Kb, however, there are 223 datapoints available, and the libscore predictions, which are based on a combinatorial peptide library, perform the best.

Next, we analyzed if the underperformance of matrix-based tools that we found when comparing in-house prediction methods could also be seen for external tools. We therefore separated the tools into matrix-based and non–matrix-based (see Materials and Methods for the classification). For 30 allele/peptide length combinations, there were predictions available from tools of both categories. We compared the highest AUC values in each tool category for all datasets. Using a paired *t* test and a Wilcoxon signed-rank test, we found that the matrix-based tools significantly outperformed the non–matrix-based tools (both with *p* < 0.05). This comparison again has to be interpreted with caution, as the access to data of the different tools is probably a much more important factor in determining prediction quality then the underlying algorithm used.

### A Web-Based Framework for the Generation and Evaluation of Prediction Methods and Tools

When evaluating our three prediction methods, we encountered multiple problems caused by differences in their implementation. All have been implemented in different programming languages: the ANN method is implemented in Fortran, SMM in C++, and ARB in Java. Also, all have different input and output requirements. It became clear that an abstraction layer providing a common interface to prediction tools and methods would be highly beneficial.

**Figure 5:**
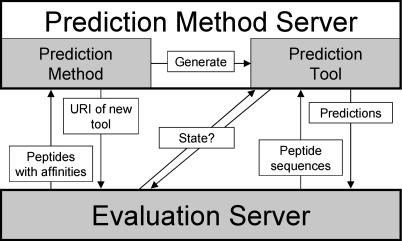
Scheme to Integrate Prediction Methods Shown is a prediction framework providing a common interface to different prediction methods to generate new tools and retrieve predictions from them. A prediction method has to accept a set of peptides with measured affinities with which it can train a new prediction tool. It returns the URI of the new tool to the evaluation server. Using the URI, the evaluation server can check for the state of the new tool to see if training is still ongoing or if an error occurred during training. Once the tool training is completed, it has to accept a set of peptide sequences and return predicted affinities for them. The format for the data exchanged in each of these steps is defined in an xml schema definition (.xsd file), available at http://mhcbindingpredictions.immuneepitope.org.

As many tools were already implemented as web servers, it was natural to define this abstraction layer as a set of http commands. We defined such a common interface to both query existing prediction tools as well as coordinate the generation of tools by prediction methods. Figure 5 gives an overview of the interactions defined in the abstraction layer.

The framework is designed to be expandable and place minimum requirements on the implementation of outside prediction methods. This will allow tool developers to plug their existing or newly developed prediction methods into the same framework for a transparent, automated comparison with other predictions. This allows controlling for both the training and testing data used, enabling a true side-by-side comparison. Also, all methods implemented this way automatically benefit from increases in the data available to the IEDB.

## Discussion

In the present report, we make available what is to date the largest dataset of quantitative peptide-binding affinities for MHC class I molecules. Establishing this dataset is part of the IEDB [39] effort, and was generated specifically with tool developers in mind. While the main part of the IEDB is structured to store a large amount of detailed immunological data, the present dataset is a curated, more homogeneous subset. This allows computer scientists and bioinformaticians to focus on improving prediction algorithms while avoiding common problems in data assembly from the literature such as inconsistent annotation of MHC alleles, handling conflicting data from unrelated assays, errors due to manual entry of the data, and, of course, the effort involved in collecting the data.

Another significant problem in the generation of peptide-MHC binding datasets is that immunologists often consider negative binding data as not interesting enough for publication. This biases the immunological literature to report only positive binding data, and forces tool developers to approximate negative binders with randomly generated peptides. While the use of random peptides is often necessary, previous studies have shown that the use of true nonbinding peptides allows for the generation of better predictions [22,49]. The present set of peptide-binding data removes the need for randomized peptides, as all binding data generated is reported, including plenty of nonbinding peptides.

The data in our set come exclusively from two assay systems established in the Buus and Sette labs. This makes it much more homogeneous than other available datasets, typically curated from the literature. Moreover, we conducted a set of reference experiments to standardize the quantitative affinities observed in the two assays. This showed that for peptides with IC_50_ values > 400 nM, the measurements of the two assays corresponded very well, less so for high-affinity peptides. We originally had hoped to convert IC_50_ values from different sources onto a common scale. However, our analysis suggests that this may not be possible due to differences in sensitivities between the two assay systems. Still, by documenting incompatibilities between assays, these can be taken into account by tool developers. Specifically for the current dataset, we recommend evaluating prediction performance by the ability to classify peptides into binders and nonbinders at a cutoff of 500 nM. We plan to include data from additional sources to this dataset, for which we will carry out a similar process of exchanging peptides and reagents to ensure consistency of the reported affinities.

We have used the dataset to evaluate the prediction performance of three methods that are routinely used by our groups. In this comparison, the ANN method outperformed the two matrix-based predictions ARB and SMM, independent of the size of the training dataset. This surprising result indicates that the primary reason for the superior ANN performance is not its ability to model higher-order sequence correlations, which would result in a larger performance gap for increasing dataset size. This does not imply that higher-order sequence correlations play no role in peptide binding to MHC. Indeed, this is very unlikely, as the peptide must fit into the binding cleft, which is restricted by the available space and contact sites, for which neighboring residues will compete. To directly assess the importance of higher-order correlations, one would need to calculate, for instance, the mutual information by estimating amino acid pair frequencies for the 400 possible pairs at two positions in the peptide [50]. However, the signal-to-noise ratio of such a calculation is still too low for datasets of the size utilized in this study, which are still very small compared to other fields where higher-order correlations definitely do play a role (e.g., secondary structure predictions).

The high performance of the ANN method on small datasets is likely due to the fact that the present ANN method being utilized is a hybrid, where the peptide amino acid sequence is represented according to several different encoding schemes, including conventional sparse encoding, Blosum encoding, and hidden Markov model encoding [41]. This encoding enables the network to generalize the impact on binding of related amino acids.

Multiple comparisons of tool prediction performance have been made before with conflicting outcomes when comparing matrix predictions with neural networks [12,38,48]. The comparison presented here is different in two main aspects. First, the magnitude of data used in this comparison is 10- to 100-fold larger than previous attempts. Second, the three methods in the comparison were all used and optimized as implemented by their developers. This avoids expert bias (i.e., the effect that a tool developer is better able to optimize predictions of methods he/she is familiar with than those he/she is unfamiliar with).

We have also evaluated the performance of external prediction tools on this dataset. As could be expected simply because of differences in the type and amount of data available to the external tools for training, their prediction performance is usually below that recorded by the methods in cross-validation. Specifically, as the set of peptide sequences was not homology-reduced, the performance of the three internal prediction methods is overestimated compared to the external tools. Therefore, we expect that the performance of all external tools will improve significantly when retraining them with the data made available here. Still, for a number of datasets, the best external predictions outperform all three methods tested in cross-validation here. In most cases, these datasets are comparably small (<140 peptides), which could explain why the three methods underperformed. One exception is the H-2 K^b^ set with 223 peptides, for which the libscore predictions, which are based on characterizing MHC binding combinatorial peptide libraries, perform best. As this requires a comparatively small number of affinity measurements (20× peptide length), this underlines the value of this approach for characterizing new MHC alleles.

All of the data generated in the evaluation process, including the dataset splits and predictions generated in cross-validation, is made publicly available. These data make the evaluation process itself transparent and allow for using them as benchmarks during tool development and testing.

While everyone can work with these benchmark sets in the privacy of their own lab, we hope that promising prediction methods will be integrated into our automated tool generation and evaluation framework. This web-based framework was designed to minimize requirements on hardware and software, and it enables a transparent side-by-side comparison of prediction methods.

Results from such a side-by-side comparison will help bioinformaticians identify which features make a prediction method successful, and they can be used as a basis for further dedicated prediction contests. Importantly, such comparisons will also help immunologists find the most appropriate prediction tools for their intended use.

The present evaluation is solely concerned with the prediction of peptide binding to MHC class I molecules. Binding of a peptide is a prerequisite for recognition during an immune response. However, there are many other factors that make some binding peptides more relevant than others for a given purpose. Examples of such factors include preferring peptides that are able to bind multiple MHC alleles, preferring peptides derived from viral proteins expressed early during infection, or preferring peptides that are efficiently generated from their source protein during antigen processing. For these and other factors, we plan to provide datasets and carry out evaluations similar to the one presented here in future studies. Our overall goal is to communicate problems of immunological relevance to bioinformaticians, and to demonstrate to immunologists how bioinformatics can aid in their work.

## Materials and Methods

### Peptide-binding assay—Sette.

The MHC peptide-binding assay utilized in the Sette lab measures the ability of peptide ligands to inhibit the binding of a radiolabeled peptide to purified MHC molecules, and has been described in detail elsewhere [43,51,52]. Briefly, however, purified MHC molecules, test peptides, and a radiolabeled probe peptide are incubated for 2 d at room temperature in the presence of human B2-microglobulin and a cocktail of protease inhibitors. After the 2-d incubation, binding of the radiolabeled peptide to the corresponding MHC class I molecule is determined by capturing MHC–peptide complexes on W6/32 antibody (anti-HLA A, B, and C)–coated plates, and measuring bound cpm using a microscintillation counter. Alternatively, following the 2-d incubation, the percent of MHC-bound radioactivity can be determined by size exclusion gel filtration chromatography. For competition assays, the concentration of peptide yielding 50% inhibition of the binding of the radiolabeled peptide is calculated. Peptides are typically tested at six different concentrations covering a 100,000-fold dose range, and in three or more independent assays. Under the conditions utilized, where [label] < [MHC] and IC_50_ ≥ [MHC], the measured IC_50_ values are reasonable approximations of the true K_D_ values [48,53].

### Peptide-binding assay—Buus.

The denatured and purified recombinant HLA heavy chains were diluted into a renaturation buffer containing HLA light chain, B2-microglobulin, and graded concentrations of the peptide to be tested, and incubated at 18 °C for 48 h allowing equilibrium to be reached. We have previously demonstrated that denatured HLA molecules can fold efficiently de novo, but only in the presence of appropriate peptide. The concentration of peptide–HLA complexes generated was measured in a quantitative enzyme-linked immunosorbent assay and plotted against the concentration of peptide offered. Since the effective concentration of HLA (3–5 nM) used in these assays is below the KD of most high-affinity peptide–HLA interactions, the peptide concentration leading to half-saturation of the HLA is a reasonable approximation of the affinity of the interaction. An initial screening procedure was employed whereby a single high concentration (20,000 nM) of peptide was incubated with one or more HLA molecules. If no complex formation was found, the peptide was assigned as a nonbinder to the HLA molecule(s) in question; conversely, if complex formation was found in the initial screening, a full titration of the peptide was performed to determine the affinity of binding.

### ARB, ANN, and SMM predictions.

The three prediction methods used in the cross-validation were applied as previously published, with all options set to their default values unless stated otherwise in the following. For the ARB method [5], two options to determine IC_50_ values exist, of which the “linear” option was chosen. For the SMM predictions [42], it is possible to predict higher-order correlations using “pair coefficients.” This option was turned off, as this led to unacceptably long calculation times for the larger datasets. The ANN method was used as described in [41].

### Prediction retrieval from external tools.

We identified MHC class I prediction tools through literature searches, and the IMGT link list at http://imgt.cines.fr/textes/IMGTbloc-notes/Immunoinformatics.html#tooMHCbp. Identical tools appearing on multiple websites—most often in combination with proteasomal cleavage/TAP transport predictions—were only included once. Several tools were not available at the time of the study (October/November 2005). One server containing multiple prediction tools (http://www.imtech.res.in/raghava/) could unfortunately not be included, as its terms of use limit the number of predictions to 20 a day.

Several tools allowed making predictions with different algorithms. In cases like this, we retrieved predictions for both, and treated them as separate tools: multipred provides predictions based on either an artificial neural network or a hidden Markov model, which we refer to as multipredann and multipredhmm. Similarly, netmhc provides neural network–based predictions (netmhc_ann) and matrix-based predictions (netmhc_matrix), and mappp provides predictions based on bimas (mapppB) and syfpeithi (mapppS) matrices.

For each tool, we mapped the MHC alleles for which predictions could be made to the four-digit HLA nomenclature (e.g., HLA-A*0201). If this mapping could not be done exactly, we left that allele–tool combination out of the evaluation. For example, HLA-A2 could refer to HLA-A*0201, A*0202, and A*0203, which do have a distinct binding specificity.

For each tool in the evaluation, we wrote a python script wrapper to automate prediction retrieval. The retrieved predictions were stored in a MySQL database. If a tool returns a nonnumeric score such as “–” to indicate nonbinding, an appropriate numeric value indicating nonbinding on the scale of the tool was stored instead.

The algorithms underlying each tool fall in the following categories: arbmatrix, bimas, hla_a2_smm, hlaligand, libscore, mapppB, mapppS, mhcpathway, mhcpred, netmhcmatrix, predbalbc, predep, rankpep, and syfpeithi are based on positional scoring matrices, while multipredann and netmhcann are based on ANNs, multipredhmm is based on a hidden Markov model, pepdist is based on a peptide–peptide distance function, and svmhc is based on a support vector machine. With two exceptions, the tools were generated based on data of peptides binding to or being eluted from individual MHC molecules. The first exception is libpred, which was generated using binding data of combinatorial peptide libraries to MHC molecules, and predep, where the 3-D structure of the MHC molecules was used to derive scoring matrices. References with more detailed description of each tool are indicated in the text.

### ROC curves.

ROC [54] curves were used to measure the ability of predictions to classify peptides into binders (experimental IC_50_ < 500 nM) or nonbinders (experimental IC_50_ ≥ 500 nM). Given a cutoff for the predicted value, predictions for peptides were separated into positive and negative subsets, allowing for calculation of the number of true-positive and false-positive predictions. Plotting the rates of true-positive predictions as a function of the rate of false-positive predictions gives an ROC curve.

Calculating the AUC provides a highly useful measure of prediction quality, which is 0.5 for random predictions and 1.0 for perfect predictions. The AUC value is equivalent to the probability that the predicted score for a randomly chosen binding peptide is (better) than that of a randomly chosen peptide that is not a binder. To assess if the AUC value of one prediction is significantly better than that of another prediction, we resampled the set of peptides for which predictions were made. Using bootstrapping with replacement, 50 new datasets were generated with a constant ratio of binder to nonbinder peptides. We then calculated the difference in AUC for the two predictions on each new dataset. One prediction was considered significantly better than another if the distribution of the AUC values was significantly different, which we measured using a paired *t* test.

## Supporting Information

Table S1 Complete List of AUC Values (76 KB XLS)

## Abbreviations

ANN: artificial neural network
ARB: average relative binding
AUC: area under the ROC curve
IEDB: Immune Epitope Database
MHC: major histocompatibility complex
ROC: receiver operating characteristic
SMM: stabilized matrix method
TAP: transporter associated with antigen presentation
